# Associations of nutrition knowledge and healthy lifestyle with body mass index status among medical students: a cross-sectional study

**DOI:** 10.3389/fnut.2026.1893421

**Published:** 2026-09-10

**Authors:** Haibo Jin, Xinyu Zhang, Mei Wang, Yuming He, Liu Yang, Yueqian Zhu

**Affiliations:** 1 School of Basic Medical Sciences and School of Public Health, Faculty of Medicine, Yangzhou University, Yangzhou, Jiangsu, China; 2 Jiangsu Key Laboratory of Integrated Traditional Chinese and Western Medicine for Prevention and Treatment of Senile Disease, Yangzhou University, Yangzhou, Jiangsu, China; 3 Affiliated Hospital of Yangzhou University, Yangzhou, Jiangsu, China; 4 Center for Disease Control and Prevention, Yangzhou, Jiangsu, China

**Keywords:** body mass index, healthy lifestyle score, medical students, multinomial logistic regression, nutrition knowledge, random forest

## Abstract

**Objectives:**

To examine the associations of nutrition knowledge and healthy lifestyle score (HLS) with body mass index (BMI) status among medical students.

**Methods:**

This cross-sectional study included 1,040 medical students from Yangzhou University, China. Participants were recruited using convenience sampling and completed an online questionnaire. BMI was calculated using self-reported height and weight and classified as underweight, normal weight, or overweight/obesity. Lifestyle factors included smoking status, alcohol consumption, physical activity, sedentary time, and sleep duration. HLS was constructed based on five healthy lifestyle factors and categorized as 0–1, 2, 3, and 4–5. Nutrition knowledge was classified as inadequate or adequate according to the questionnaire score. Multinomial logistic regression was used to examine the associations of individual lifestyle factors, nutrition knowledge, and HLS with BMI status, using normal weight as the reference category. Exploratory indirect association analysis and random forest analysis were further conducted.

**Results:**

Among the 1,040 participants, 240 (23.1%) were classified as underweight, 540 (51.9%) as normal weight, and 260 (25.0%) as overweight/obesity. After adjustment for sex and age group, adequate nutrition knowledge was associated with lower odds of both underweight (*OR* = 0.145, 95% *CI*: 0.103–0.204) and overweight/obesity (*OR* = 0.087, 95% *CI*: 0.060–0.126). Higher HLS categories were also associated with lower odds of both underweight and overweight/obesity. Compared with participants with an HLS score of 0–1, those with an HLS score of 4–5 had lower odds of underweight (*OR* = 0.116, 95% *CI*: 0.061–0.222) and overweight/obesity (*OR* = 0.040, 95% *CI*: 0.019–0.088). Exploratory indirect association analysis suggested that HLS accounted for part of the statistical association between nutrition knowledge and BMI status. Random forest analysis further identified nutrition knowledge and lifestyle-related variables as important contributors to BMI classification.

**Conclusion:**

Nutrition knowledge and healthy lifestyle patterns were associated with BMI status among medical students. Higher HLS and adequate nutrition knowledge were associated with lower odds of both underweight and overweight/obesity. Given the cross-sectional design, these findings should be interpreted as associations rather than evidence of causality. Future longitudinal studies are needed to clarify the temporal relationships among nutrition knowledge, lifestyle behaviors, and BMI status.

## 1 Background

In recent years, the increasing prevalence of overweight and obesity has become a major public health concern. Elevated body mass index (BMI) is associated with a higher burden of cardiovascular disease, diabetes, and other chronic conditions (1, 2). In China, overweight and obesity have also become important public health challenges (3). According to the Report on Nutrition and Chronic Disease Status of Chinese Residents (2020), the prevalence of overweight and obesity among Chinese adults aged 18 years and older was 34.3 and 16.4%, respectively (4). In 2024, the National Health Commission of China launched the “Year of Weight Management” campaign to improve public awareness, promote healthy lifestyles, and strengthen weight management services (5). Against this public health and policy background, university students represent an important population for weight-related health assessment because lifestyle behaviors and nutrition-related cognition are formed and consolidated during early adulthood.

Lifestyle-related behaviors are closely related to BMI status among university students. Recent studies in university populations have shown that BMI differs across lifestyle patterns, including physical activity, dietary habits, sleep, alcohol consumption, and other health-related behaviors (6, 7). Saintila et al. (6) reported differences in BMI and healthy lifestyle practices among Peruvian university students from different academic disciplines. Bakarman et al. (7) further highlighted the relevance of obesity-related knowledge, perception, dietary habits, and physical activity among undergraduate healthcare students. These findings suggest that BMI status in university students should be examined in relation to multiple lifestyle-related characteristics rather than a single behavior alone.

Nutrition knowledge is another important factor in weight-related health assessment. It reflects students’ understanding of dietary recommendations, food choices, nutrition-related diseases, and food safety. Recent studies have shown that nutrition knowledge among university students varies by demographic and educational characteristics and is associated with eating attitudes and dietary behaviors (8, 9). Deng et al. (8) examined demographic variation in nutrition knowledge and its relationship with eating attitudes among Chinese university students, while Belogianni et al. (9) reported variations in nutrition knowledge among university students in the United Kingdom. In addition, a recent cross-sectional study among Turkish adults showed that weight management-related nutrition knowledge, physical activity, and dietary patterns were interrelated (10). These studies support the relevance of examining nutrition knowledge together with lifestyle-related behaviors in weight-related health research.

Despite growing evidence on lifestyle behaviors and nutrition knowledge, several gaps remain. First, many studies have focused on individual lifestyle factors, whereas fewer have examined a composite healthy lifestyle score that integrates smoking, alcohol consumption, physical activity, sedentary time, and sleep duration. Second, nutrition knowledge and lifestyle behaviors are often examined separately, although both cognitive and behavioral factors are relevant to BMI status. Third, previous studies frequently focus on overweight or obesity, while underweight is less often distinguished from overweight/obesity. Combining underweight and overweight/obesity into a single abnormal BMI category may obscure different association patterns. Evidence on combined healthy lifestyle factors and obesity also supports the need to evaluate lifestyle patterns as a whole (11).

Therefore, this study aimed to examine the associations of nutrition knowledge and healthy lifestyle score with BMI status among medical students. BMI status was classified as underweight, normal weight, and overweight/obesity. In addition, exploratory indirect association analysis was conducted to assess whether HLS accounted for part of the statistical association between nutrition knowledge and BMI status, and exploratory random forest analysis was used to assess BMI classification and variable importance.

## 2 Participants and methods

### 2.1 Participants

This cross-sectional study was conducted among medical students at Yangzhou University, China. Participants were recruited using convenience sampling. Students from the first to fourth academic years were invited to complete an online questionnaire. The study did not use a probability sampling framework; therefore, the sample should be interpreted as a convenience survey sample of medical students from one university rather than a probability sample. A total of 1,138 questionnaires were collected. After excluding invalid questionnaires, including those with missing key information or implausible responses, 1,040 participants were included in the final analysis, with a valid questionnaire rate of 91.39%.

Eligible participants were full-time medical students at Yangzhou University who were willing to participate and provided informed consent. Participants with incomplete information on height, weight, lifestyle factors, nutrition knowledge, or other key variables were excluded from the analysis.

## 3 Methods

### 3.1 Demographic and anthropometric information

An online self-administered questionnaire was developed to collect demographic characteristics, including sex, ethnicity, date of birth, height, and weight. Body mass index (BMI) was calculated as weight (kg)/height (m^2^). According to the Guidelines for the Prevention and Control of Overweight and Obesity in Chinese Adults, BMI was categorized into three groups according to the Chinese adult BMI classification: underweight (BMI < 18.5 kg/m^2^), normal weight (18.5 ≤ BMI < 24.0 kg/m^2^), and overweight/obesity (BMI ≥ 24.0 kg/m^2^) (12). Normal weight was used as the reference category in multinomial logistic regression analyses.

### 3.2 Lifestyle assessment

Lifestyle factors were assessed using (1) a self-designed questionnaire covering smoking, alcohol consumption, physical activity, sedentary behavior, and sleep, and (2) the Chinese Residents Aged 18–64 Years Nutrition and Health Knowledge Questionnaire compiled by the Chinese Center for Disease Control and Prevention (13). The complete questionnaire is presented in the appendix. Operational definitions were as follows:

*Smoking*: Current smoking was defined as smoking within the past 30 days and categorized as unhealthy; non-smoking was defined as healthy.

*Alcohol consumption*: Any alcohol intake in the past 12 months was classified as current drinking (unhealthy); otherwise, non-drinking was classified as healthy.

*Physical activity*: Weekly duration of moderate- to vigorous-intensity exercise < 4 h was considered unhealthy, whereas ≥ 4 h was considered healthy (14).

*Sedentary behavior*: Total time spent sitting, reclining, or lying (excluding sleep) during a typical day—including studying, reading, watching television, computer use, and rest—was assessed. Sedentary time ≥ 8 h/day was considered unhealthy; < 8 h/day was considered healthy (15).

*Sleep duration*: Total daily sleep time (including naps) < 6 h was defined as unhealthy; ≥ 6 h was defined as healthy (16).

Each lifestyle factor was dichotomized (1 = healthy, 0 = unhealthy). Scores were summed to calculate the Healthy Lifestyle Score (HLS), ranging from 0 to 5, with higher scores indicating a healthier lifestyle. Participants were categorized into four groups based on HLS: 0–1, 2, 3, and 4–5.

### 3.3 Nutrition and health knowledge

Nutrition knowledge was measured using the above-mentioned questionnaire, which consists of five domains: dietary recommendations (28 points), food and nutrients (19 points), nutrition and disease (26 points), food choices (11 points), and food safety (16 points). The questionnaire includes multiple-choice and single-choice items. Correct answers to single-choice items were awarded 1–1.5 points, while multiple-choice items were worth 4–6 points each. The total score ranged from 0 to 100, with ≥ 75 points defined as adequate nutrition knowledge (healthy) and < 75 points defined as inadequate (unhealthy) (17). The scoring criteria are summarized in Supplementary Table S1.

### 3.4 Quality control

Data were collected using an online questionnaire. After data collection, responses were entered into a database using EpiData software with double-entry verification. Questionnaires with responses that were logically inconsistent or clearly contradicted objective facts were excluded as invalid.

### 3.5 Covariates

Sex and age group were included as covariates in the adjusted models. Ethnicity and academic year were described in the baseline characteristics but were not included in the main adjusted regression models because of sparse categories and potential overlap between academic year and age group. Other potential confounders, including socioeconomic status, place of residence, detailed dietary intake, psychological status, chronic disease history, specific major, and class-level factors, were not available in the dataset; therefore, residual confounding could not be fully excluded.

### 3.6 Statistical analysis

Continuous variables were summarized as mean and standard deviation or median and interquartile range, as appropriate. Categorical variables were summarized as frequencies and percentages. Baseline characteristics were compared across BMI categories using chi-square tests or Fisher’s exact tests, as appropriate.

Multinomial logistic regression models were used to examine the associations of individual lifestyle factors, nutrition knowledge, and HLS with BMI status. Normal weight was used as the reference BMI category. Odds ratios (*OR*s) and 95% confidence intervals (*CI*s) were calculated for underweight versus normal weight and overweight/obesity versus normal weight. Both unadjusted and adjusted models were fitted. The adjusted models included sex and age group.

An exploratory indirect association analysis was conducted to examine whether HLS accounted for part of the statistical association between nutrition knowledge and BMI status. Nutrition knowledge was treated as the exposure, HLS as the intermediate variable, and BMI status as the outcome. The analysis was conducted separately for underweight versus normal weight and overweight/obesity versus normal weight. Associations were estimated on the log-odds scale and then exponentiated as *OR*s. The proportion accounted for was calculated on the log-odds scale. Given the cross-sectional design, this analysis was interpreted as an exploratory statistical analysis rather than evidence of causal mediation.

Exploratory random forest models were used to assess BMI classification performance and variable importance. For the random forest analysis, the dataset was partitioned into a training set and a testing set at a ratio of 70:30. Five-fold cross-validation was performed in the training set to tune the mtry parameter, and 500 trees were fitted in each random forest model. Two models were constructed. Model A included sex, age group, individual lifestyle components, and nutrition knowledge. Model B included sex, age group, HLS group, and nutrition knowledge. Model performance was evaluated in the testing set using accuracy, Kappa, multiclass area under the receiver operating characteristic curve (AUROC), macro area under the precision-recall curve (AUPRC), and out-of-bag error. Class-specific performance metrics and calibration curves were also examined. Variable importance was assessed using MeanDecreaseAccuracy and MeanDecreaseGini. The random forest models were used for exploratory classification and variable importance analysis rather than for clinical prediction.

All statistical analyses were performed using R version 4.4.2. A two-sided *p*-value <0.05 was considered statistically significant.

## 4 Results

### 4.1 Baseline characteristics of participants

A total of 1,040 participants were included in the final analysis, including 240 underweight participants (23.1%), 540 normal-weight participants (51.9%), and 260 participants with overweight/obesity (25.0%).

As shown in Table 1, sex, age group, smoking status, alcohol consumption, physical activity, sedentary time, sleep duration, nutrition knowledge, and HLS category differed significantly across BMI categories. Ethnicity showed no significant difference among the three BMI groups. Compared with participants with normal weight, those with underweight or overweight/obesity generally showed less favorable distributions of lifestyle-related factors, nutrition knowledge, and HLS category.

**Table 1:** Baseline characteristics of participants by BMI status (*N* = 1,040).

Variable	BMI			*p-*value
	Normal^a^ (*n* = 540)	Underweight^b^ (*n* = 240)	Overweight/obesity^c^ (*n* = 260)	
Sex, *n* (%)				
Male	182 (33.70)	92 (38.33)	124 (47.69)	< 0.001
Female	358 (66.30)	148 (61.67)	136 (52.31)	
Ethnicity, *n* (%)				
Han	515 (95.37)	229 (95.42)	247 (95.00)	0.962
Other	25 (4.63)	11 (4.58)	13 (5.00)	
Age, *n* (%)				
18-22 years	519 (96.11)	221 (92.08)	233 (89.62)	< 0.001
< 18 years	13 (2.41)	2 (0.83)	3 (1.15)	
> 22 years	8 (1.48)	17 (7.08)	24 (9.23)	
Smoking status, *n* (%)				
Smoker	4 (0.74)	4 (1.67)	17 (6.54)	< 0.001
Non-smoker	536 (99.26)	236 (98.33)	243 (93.46)	
Alcohol consumption, *n* (%)				
Drinker	256 (47.41)	188 (78.33)	178 (68.46)	< 0.001
Non-drinker	284 (52.59)	52 (21.67)	82 (31.54)	
Physical activity, *n* (%)				
<4 h/week	312 (57.78)	165 (68.75)	221 (85.00)	< 0.001
≥4 h/week	228 (42.22)	75 (31.25)	39 (15.00)	
Sedentary time, *n* (%)				
≥8 h/day	253 (46.85)	152 (63.33)	132 (50.77)	< 0.001
<8 h/day	287 (53.15)	88 (36.67)	128 (49.23)	
Sleep duration, *n* (%)				
<6 h/day	279 (51.67)	156 (65.00)	198 (76.15)	< 0.001
≥6 h/day	261 (48.33)	84 (35.00)	62 (23.85)	
Nutrition knowledge, *n* (%)				
< 75 score	128 (23.70)	166 (69.17)	206 (79.23)	< 0.001
≥ 75 score	412 (76.30)	74 (30.83)	54 (20.77)	
Healthy lifestyle score (HLS), *n* (%)				
0–1 points	39 (7.22)	42 (17.50)	55 (21.15)	< 0.001
2 points	116 (21.48)	120 (50.00)	126 (48.46)	
3 points	234 (43.33)	58 (24.17)	69 (26.54)	
4–5 points	151 (27.96)	20 (8.33)	10 (3.85)	
Total	540(51.92)	240(23.08)	260(25.00)	-

a Normal BMI was defined as 18.5 ≤ BMI < 24.0 kg/m^2^ according to the Chinese adult BMI classification.

b Underweight was defined as BMI < 18.5 kg/m^2^ according to the Chinese adult BMI classification.

c Overweight/Obesity (BMI ≥ 24.0 kg/m^2^) according to the Chinese adult BMI classification.

These baseline differences suggested that demographic and lifestyle-related characteristics varied across BMI status groups, and these variables were further examined in the subsequent association analyses.

### 4.2 Associations of individual lifestyle factors and nutrition knowledge with BMI status

**Figure 1:**
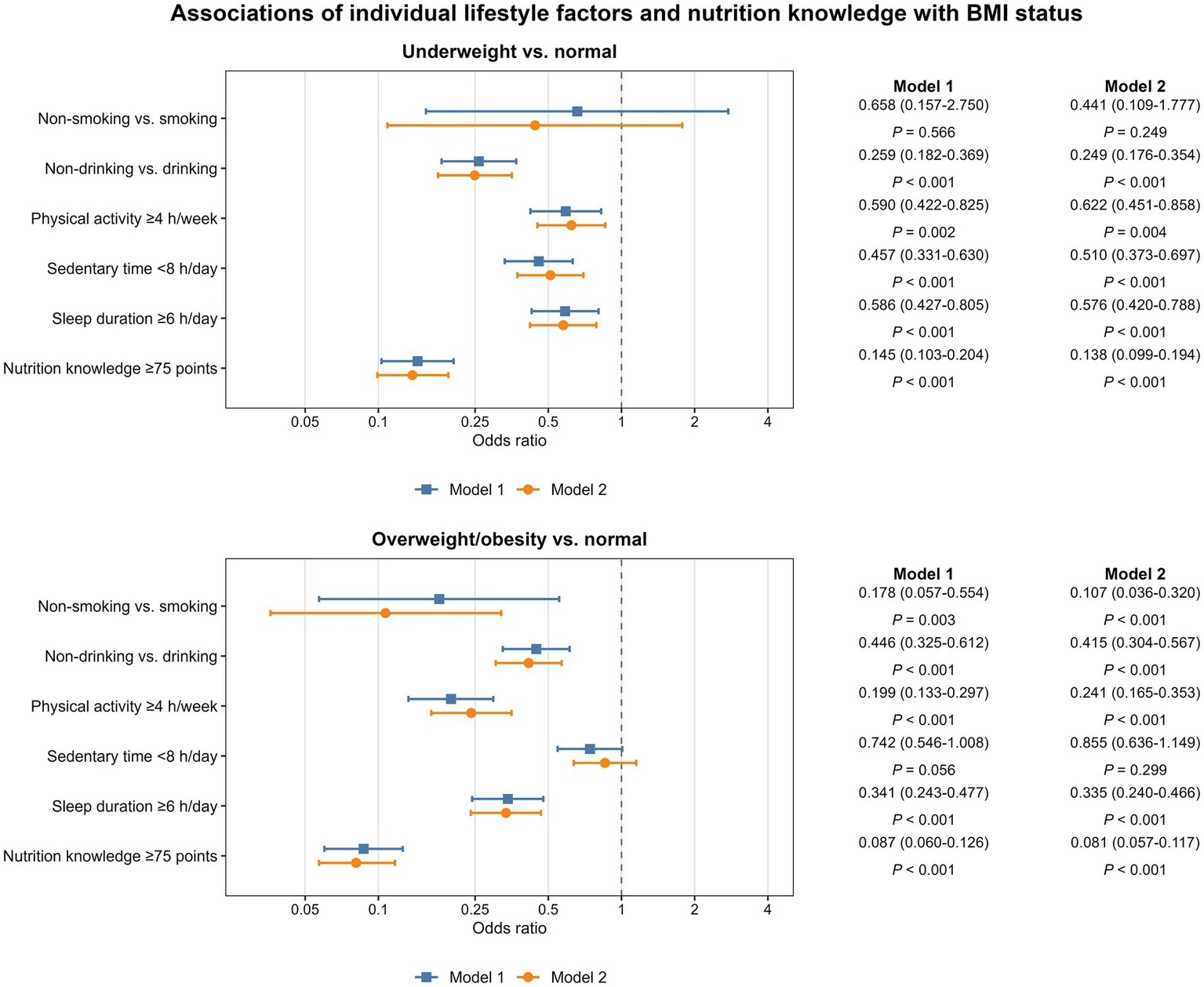
Associations of individual lifestyle factors and nutrition knowledge with BMI status. Odds ratios and 95% confidence intervals were estimated using multinomial logistic regression, with normal weight as the reference category. Model 1 was adjusted for sex and age group; Model 2 was unadjusted. OR, odds ratio; CI, confidence interval.

Using normal weight as the reference BMI category, the associations of individual lifestyle factors and nutrition knowledge with underweight and overweight/obesity are shown in Figure 1 and Supplementary Table S2. After adjustment for sex and age group, non-smoking was associated with lower odds of overweight/obesity (*OR* = 0.178, 95% *CI*: 0.057–0.554), but its association with underweight was not statistically significant.

Non-drinking, sufficient physical activity, adequate sleep duration, and adequate nutrition knowledge were generally associated with lower odds of both underweight and overweight/obesity. Specifically, non-drinking was associated with lower odds of both underweight (*OR* = 0.259, 95% *CI*: 0.182–0.369) and overweight/obesity (*OR* = 0.446, 95% *CI*: 0.325–0.612). Physical activity ≥4 h/week was also associated with lower odds of underweight (*OR* = 0.590, 95% *CI*: 0.422–0.825) and overweight/obesity (*OR* = 0.199, 95% *CI*: 0.133–0.297). Sedentary time <8 h/day was associated with lower odds of underweight (*OR* = 0.457, 95% *CI*: 0.331–0.630), whereas its association with overweight/obesity did not reach statistical significance after adjustment (*OR* = 0.742, 95% *CI*: 0.546–1.008). Sleep duration ≥6 h/day was associated with lower odds of both underweight (*OR* = 0.586, 95% *CI*: 0.427–0.805) and overweight/obesity (*OR* = 0.341, 95% *CI*: 0.243–0.477).

Adequate nutrition knowledge showed the strongest association with BMI status. Compared with participants with inadequate nutrition knowledge, those with adequate nutrition knowledge had lower odds of underweight (*OR* = 0.145, 95% *CI*: 0.103–0.204) and overweight/obesity (*OR* = 0.087, 95% *CI*: 0.060–0.126). Overall, the adjusted results were generally consistent with the unadjusted analyses.

### 4.3 Associations between HLS and BMI status

**Figure 2:**
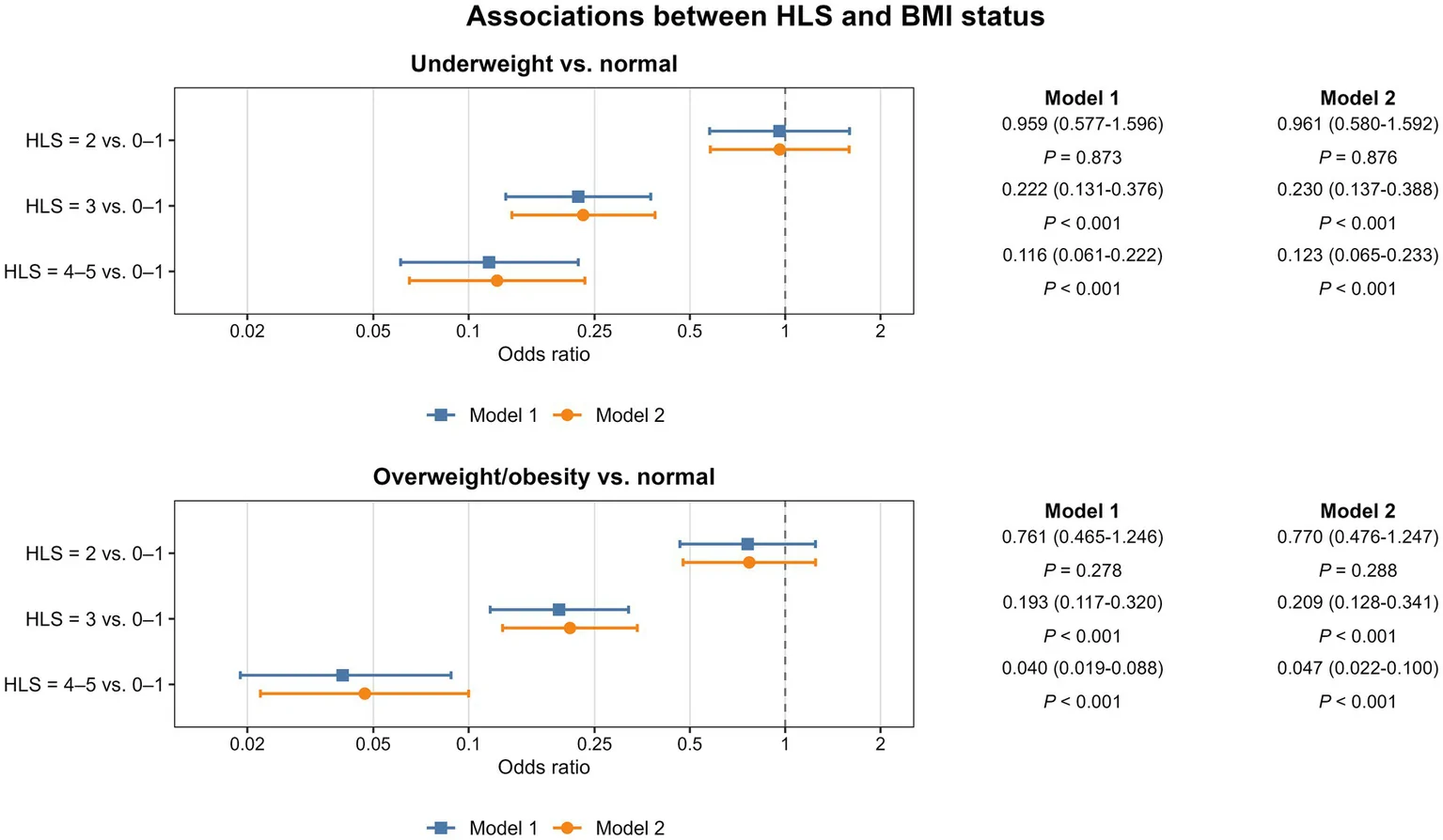
Associations between HLS and BMI status. *OR* and *95% CI* were estimated using multinomial logistic regression models, with normal weight and HLS score of 0–1 as the reference groups. Model 1 was adjusted for sex and age group; Model 2 was unadjusted.

Using normal weight as the reference BMI category and HLS score of 0–1 as the reference group, the associations between HLS and BMI status are shown in Figure 2 and Supplementary Table S3. After adjustment for sex and age group, HLS score of 2 was not significantly associated with underweight or overweight/obesity compared with the reference HLS group.

In contrast, higher HLS categories were significantly associated with lower odds of both underweight and overweight/obesity. Compared with participants with an HLS score of 0–1, those with an HLS score of 3 had lower odds of underweight (*OR* = 0.222, 95% *CI*: 0.131–0.376) and overweight/obesity (*OR* = 0.193, 95% *CI*: 0.117–0.320). The associations were stronger among participants with an HLS score of 4–5, with adjusted ORs of 0.116 (95% *CI*: 0.061–0.222) for underweight and 0.040 (95% *CI*: 0.019–0.088) for overweight/obesity.

Overall, higher HLS categories were associated with more favorable BMI status, and the adjusted results were generally consistent with the unadjusted analyses.

### 4.4 Exploratory indirect association analysis of nutrition knowledge, HLS, and BMI status

**Figure 3:**
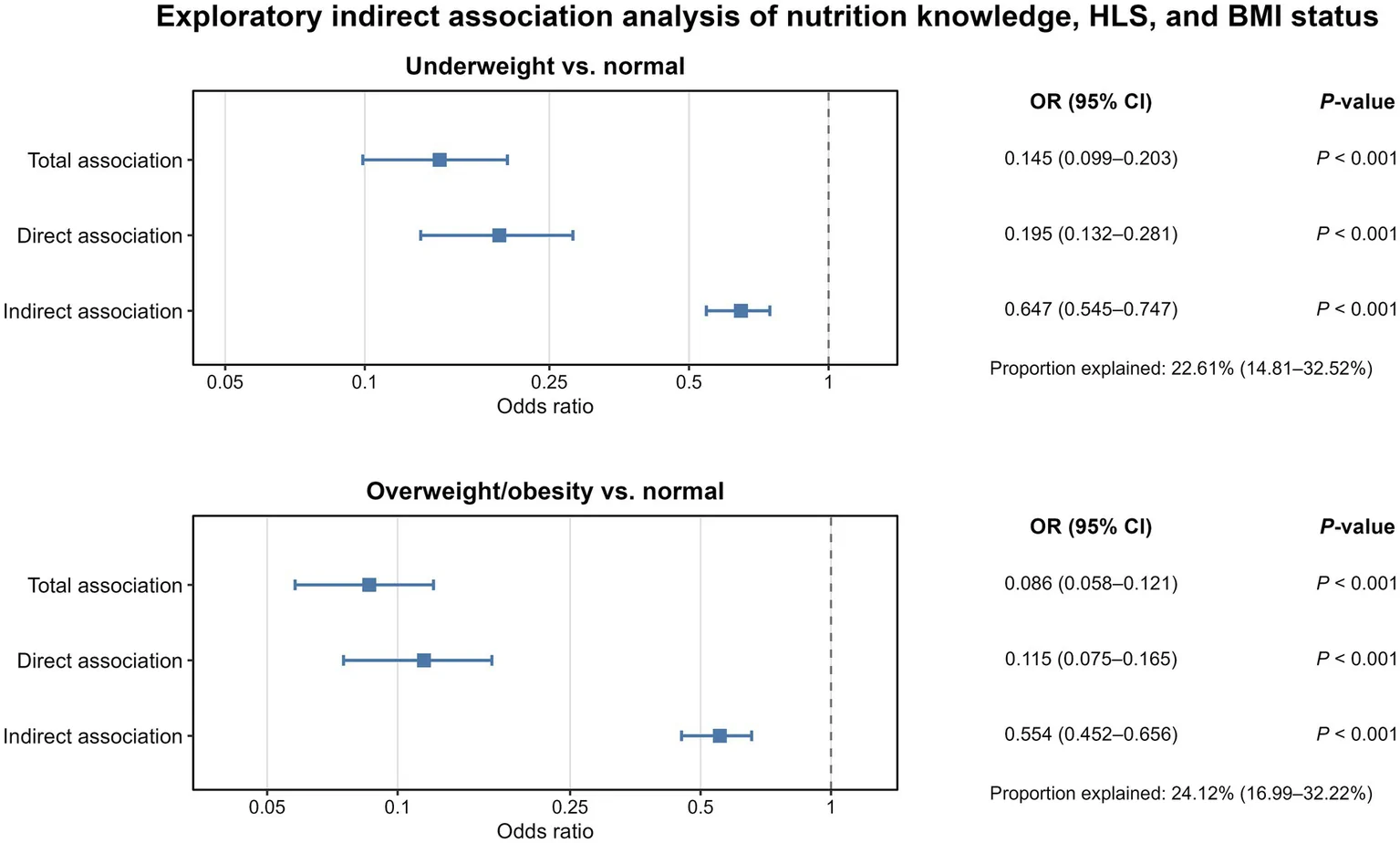
Exploratory indirect association analysis of nutrition knowledge, HLS, and BMI status. The figure presents total, direct, and indirect associations of nutrition knowledge with BMI status through HLS. Models were adjusted for sex and age group. Results should be interpreted as exploratory statistical associations rather than evidence of causal mediation.

Using normal weight as the reference BMI category, the exploratory indirect association analysis of nutrition knowledge, HLS, and BMI status is shown in Figure 3 and Supplementary Table S4. After adjustment for sex and age group, adequate nutrition knowledge was associated with lower odds of both underweight and overweight/obesity. For underweight versus normal weight, the total association *OR* was 0.145 (95% *CI*: 0.099–0.203), and the direct association *OR* was 0.195 (95% *CI*: 0.132–0.281). The indirect association through HLS was also significant, with an *OR* of 0.647 (95% *CI*: 0.545–0.747), accounting for 22.61% of the total association on the log-odds scale.

For overweight/obesity versus normal weight, the total association OR was 0.086 (95% *CI*: 0.058–0.121), and the direct association *OR* was 0.115 (95% *CI*: 0.075–0.165). The indirect association through HLS was 0.554 (95% *CI*: 0.452–0.656), accounting for 24.12% of the total association on the log-odds scale.

Overall, HLS accounted for part of the statistical association between nutrition knowledge and BMI status. Given the cross-sectional design, these findings should be interpreted as exploratory statistical associations rather than evidence of causal mediation.

### 4.5 Exploratory random forest analysis for BMI classification

Exploratory random forest models were further used to assess BMI classification performance and variable importance. As shown in Table 2, Model A, which included individual lifestyle components and nutrition knowledge, showed slightly better overall performance than Model B, which included HLS group and nutrition knowledge. Model A achieved a test accuracy of 0.660, a Kappa value of 0.425, a multiclass AUROC of 0.756, and a macro-AUPRC of 0.612. In comparison, Model B showed a test accuracy of 0.619, a Kappa value of 0.345, a multiclass AUROC of 0.712, and a macro-AUPRC of 0.552.

**Table 2:** Performance of exploratory random forest models for BMI classification.

Model	Accuracy	Kappa	Multiclass AUROC	Macro AUPRC	OOB error
Model A: lifestyle components + nutrition knowledge	0.660	0.425	0.756	0.612	0.394
Model B: HLS group + nutrition knowledge	0.619	0.345	0.712	0.552	0.396

Class-specific performance metrics are presented in Supplementary Table S5. Both models showed better classification performance for normal weight than for underweight. Calibration curves are shown in Supplementary Figure S1, suggesting relatively better calibration for normal weight and less stable calibration for underweight.

**Figure 4:**
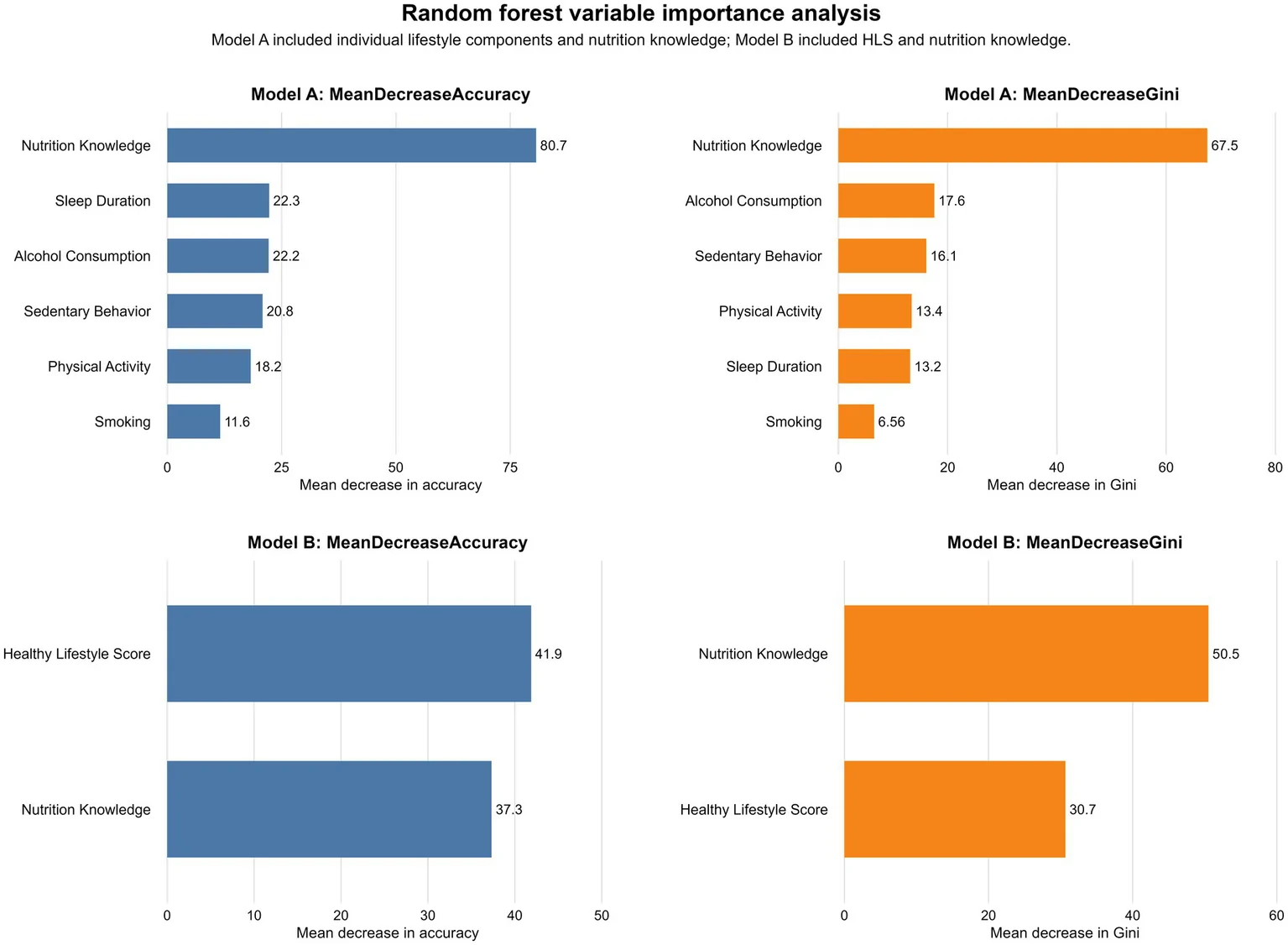
Random forest variable importance analysis for BMI classification. Variable importance was evaluated using MeanDecreaseAccuracy and MeanDecreaseGini. Model A included individual lifestyle components and nutrition knowledge. Model B included HLS group and nutrition knowledge.

Variable importance rankings are presented in Figure 4 and Supplementary Table S6. In Model A, nutrition knowledge ranked as the most important variable, followed by several lifestyle-related factors, including sleep duration, alcohol consumption, sedentary behavior, and physical activity. In Model B, nutrition knowledge remained the most important variable, while HLS group also showed notable importance. Sex-stratified variable importance analyses are shown in Supplementary Figure S2. Nutrition knowledge remained an important variable in both sexes, while the relative importance of lifestyle-related variables showed some differences between male and female participants. These findings suggest that nutrition knowledge and lifestyle-related characteristics contributed to BMI classification in the exploratory random forest models. However, because the models were not externally validated, these results should be interpreted as exploratory classification and variable importance findings rather than evidence of clinical prediction or causal importance.

## 5 Discussion

This cross-sectional study examined the associations of nutrition knowledge and healthy lifestyle score with BMI status among medical students. By distinguishing underweight from overweight/obesity, this study provides a more detailed assessment of BMI status than analyses that combine both conditions into a single abnormal BMI category. Adequate nutrition knowledge and higher HLS categories were associated with more favorable BMI status. In addition, the exploratory indirect association analysis indicated that HLS accounted for part of the statistical association between nutrition knowledge and BMI status. The exploratory random forest analysis further showed that nutrition knowledge and lifestyle-related variables contributed to BMI classification.

A central contribution of this study is the integration of nutrition knowledge with lifestyle patterns in the assessment of BMI status among medical students. Previous studies have shown that nutrition knowledge varies across student populations and is related to eating attitudes and dietary behaviors (8, 9). The present study extends this evidence by linking nutrition knowledge with both lifestyle-related characteristics and BMI categories. This framework is relevant for university populations because students are in a transitional life stage in which dietary choices, physical activity, alcohol consumption, sleep habits, and sedentary behaviors become more independent and less structured. Nutrition knowledge therefore represents a measurable cognitive factor that is closely connected with lifestyle-related health assessment in this population.

The association between nutrition knowledge and BMI status deserves specific attention. Students with adequate nutrition knowledge had more favorable BMI status than those with inadequate nutrition knowledge. This finding is consistent with studies showing that nutrition knowledge is associated with healthier eating attitudes, nutrition label use, and dietary quality (18, 19). It is also consistent with recent evidence indicating that weight management-related nutrition knowledge, physical activity, and dietary patterns are interrelated (10). However, nutrition knowledge should not be interpreted as a direct determinant of BMI in the present study. Because all variables were measured at the same time, the temporal order between knowledge, behavior, and BMI status cannot be established. A balanced interpretation is that nutrition knowledge and BMI status are linked within a broader pattern of health awareness, dietary decision-making, and lifestyle behaviors.

HLS was also associated with BMI status, supporting the value of examining combined lifestyle patterns rather than isolated behaviors alone. Previous studies have reported that adherence to multiple healthy lifestyle behaviors is associated with lower obesity prevalence or more favorable weight-related outcomes (11, 20, 21). In the present study, higher HLS categories were associated with lower odds of both underweight and overweight/obesity. This finding indicates that a composite lifestyle score captures cumulative information from multiple behaviors that are relevant to BMI status. The absence of a clear association for the HLS score of 2 further indicates that limited behavioral improvement is insufficient to distinguish BMI status clearly, whereas a broader clustering of healthy behaviors provides stronger discrimination across BMI categories.

The analysis of individual lifestyle factors further clarifies the role of specific behaviors. Non-drinking, sufficient physical activity, adequate sleep duration, and adequate nutrition knowledge were associated with lower odds of both underweight and overweight/obesity, whereas sedentary time and smoking showed more category-specific patterns. These findings indicate that underweight and overweight/obesity should not be treated as interchangeable outcomes. Underweight among medical students can reflect insufficient dietary intake, irregular routines, psychological stress, or other unmeasured factors, whereas overweight/obesity is more closely related to excess energy balance and sedentary lifestyles. Separating these BMI categories improves interpretability and directly addresses the methodological concern that combining underweight and overweight/obesity may obscure distinct associations.

The findings related to physical activity and sedentary behavior are consistent with broader evidence that movement-related behaviors are associated with weight-related outcomes, although the magnitude of association varies across populations and measurement methods. Evidence syntheses have shown that exercise training is associated with changes in anthropometric outcomes among adults with overweight or obesity, while the extent of improvement depends on exercise modality, duration, and accompanying lifestyle conditions (22, 23). Recent experimental evidence also supports the relevance of interrupting prolonged sitting with light-intensity activity for cardiometabolic health in young adults with overweight or obesity (24). In the present university sample, physical activity was associated with both underweight and overweight/obesity, whereas sedentary time was more clearly associated with underweight after adjustment. This pattern indicates that movement-related behaviors should be interpreted as part of a broader lifestyle profile rather than as isolated determinants of BMI status.

The finding related to smoking requires cautious interpretation. Non-smoking was associated with lower odds of overweight/obesity, but its association with underweight was not statistically significant. Because the number of smokers in this sample was small, the estimate may be less stable than those for other lifestyle factors. Previous research has also shown that smoking, nicotine exposure, appetite, and body weight are linked through complex behavioral and biological pathways (25). Therefore, smoking should be interpreted as one component of the broader lifestyle profile rather than as an independent explanatory factor for BMI status in this study.

The exploratory indirect association analysis adds a hypothesis-generating perspective to the study. HLS statistically explained part of the association between nutrition knowledge and BMI status, accounting for approximately one quarter of the total association on the log-odds scale. This result is compatible with a conceptual pathway in which nutrition knowledge is linked with BMI status through lifestyle-related behaviors. Nevertheless, this analysis should be interpreted as an exploratory statistical association rather than causal mediation. The cross-sectional design does not establish whether nutrition knowledge preceded lifestyle behaviors or whether BMI status influenced students’ interest in nutrition and health information. The value of this analysis lies in identifying a coherent behavioral pathway that can be tested in future longitudinal or intervention studies.

The exploratory random forest analysis provided complementary evidence. Model A, which included individual lifestyle components and nutrition knowledge, showed slightly better classification performance than Model B, which included HLS group and nutrition knowledge. This indicates that individual lifestyle components retain information that is partly compressed when they are combined into a single score. At the same time, HLS remained informative in Model B, supporting its utility as a concise summary of lifestyle patterns. Nutrition knowledge ranked highly in both models, strengthening the consistency between regression-based association analysis and machine-learning-based variable importance assessment. However, the random forest models were not externally validated and showed weaker performance for underweight than for normal weight. These findings should therefore be regarded as exploratory classification and variable importance results, not as evidence of a clinically applicable prediction model.

This study has several strengths. First, BMI status was classified into underweight, normal weight, and overweight/obesity, avoiding the loss of information caused by combining underweight with overweight/obesity. Second, both individual lifestyle factors and a composite HLS were examined, allowing comparison between behavior-specific and pattern-based associations. Third, nutrition knowledge was incorporated into the analytic framework, adding scientific value because knowledge is a modifiable cognitive factor directly relevant to campus health education. Fourth, traditional regression analysis was supplemented with exploratory indirect association analysis and random forest variable importance analysis. This multimethod strategy improves the descriptive depth of the study while maintaining a clear distinction between statistical association, exploratory pathway analysis, and classification performance.

Several limitations should be acknowledged. First, the cross-sectional design precludes conclusions about temporality or causality. The results should therefore be interpreted as associations rather than evidence that nutrition knowledge or lifestyle behaviors cause changes in BMI status. Second, height and weight were self-reported. Evidence from young adult samples indicates that self-reported height and weight can show high concordance with measured values, but reporting bias and BMI misclassification remain possible (26). Third, this study was based on a convenience sample of medical students from one university and did not use a probability sampling framework, which limits generalizability to students from other disciplines, universities, or regions. Fourth, although the adjusted models included sex and age group, several potential confounders, including socioeconomic status, faculty or department, place of residence, detailed dietary intake, psychological status, and chronic disease history, were not available. Residual confounding therefore cannot be excluded. Finally, the random forest models were exploratory and lacked external validation; their results should not be used for clinical prediction.

In conclusion, this study provides evidence that nutrition knowledge and healthy lifestyle patterns are associated with BMI status among medical students. The findings highlight the importance of considering both cognitive and behavioral factors when examining BMI status in this population. The separation of underweight from overweight/obesity, together with the integration of HLS, exploratory indirect association analysis, and random forest variable importance, contributes to a more nuanced understanding of weight-related health profiles among medical students. Future longitudinal studies with objective anthropometric measurements, more comprehensive covariate information, and repeated assessments of nutrition knowledge and lifestyle behaviors are needed to clarify temporal relationships and evaluate whether campus-based nutrition and lifestyle programs are associated with changes in BMI status.

## 6 Conclusion

In this cross-sectional study of medical students, nutrition knowledge and healthy lifestyle score were associated with BMI status. Adequate nutrition knowledge and higher HLS categories were associated with lower odds of both underweight and overweight/obesity compared with normal weight. The exploratory indirect association analysis suggested that HLS accounted for part of the statistical association between nutrition knowledge and BMI status. In addition, exploratory random forest analysis indicated that nutrition knowledge and lifestyle-related factors contributed to BMI classification.

These findings highlight the potential relevance of nutrition knowledge and healthy lifestyle patterns in relation to BMI status among medical students. Given the cross-sectional design and the use of self-reported height and weight, the results should be interpreted as associations rather than evidence of causality. Future longitudinal studies are needed to clarify the temporal relationships among nutrition knowledge, lifestyle behaviors, and BMI status. In addition, exploratory random forest analysis indicated that nutrition knowledge and lifestyle-related factors contributed to BMI classification.

## Data Availability

The raw data supporting the conclusions of this article will be made available by the authors, without undue reservation.

## Supplementary material

SUPPLEMENTARY FIGURE S1 Calibration curves of exploratory random forest models. Calibration curves show the observed proportions against the mean predicted probabilities across probability bins for each BMI class. The dashed diagonal line represents perfect calibration. Model A included individual lifestyle components and nutrition knowledge, whereas Model B included HLS group and nutrition knowledge. The models were used for exploratory classification and variable importance analysis rather than clinical prediction.

SUPPLEMENTARY FIGURE S2 Sex-stratified random forest variable importance analysis. Variable importance was assessed separately among male and female participants using exploratory random forest models. Importance was evaluated using MeanDecreaseAccuracy and MeanDecreaseGini. Model A included individual lifestyle components and nutrition knowledge, whereas Model B included HLS group and nutrition knowledge. The figure was used to compare the relative importance of nutrition knowledge and lifestyle-related variables across sex strata. These analyses were exploratory and should not be interpreted as evidence of causal importance or clinical prediction.

## References

[1] Roth GA Mensah GA Johnson CO Addolorato G Ammirati E Baddour LM . Global burden of cardiovascular diseases and risk factors, 1990–2019: update from the GBD 2019 study. J Am Coll Cardiol. (2020) 76:2982–3021. doi: 10.1016/j.jacc.2020.11.010, 33309175 PMC7755038

[2] Zhang X Wang X Wang M Hu B Tang W Wu Y . The global burden of type 2 diabetes attributable to high body mass index in 204 countries and territories, 1990-2019: an analysis of the global burden of disease study. Front Public Health. (2022) 10:966093. doi: 10.3389/fpubh.2022.966093, 36159296 PMC9500174

[3] Xiang H Yang R Tu J Guan X Tao X. Health impacts of high BMI in China: terrible present and future. Int J Environ Res Public Health. (2022) 19:16173. doi: 10.3390/ijerph192316173, 36498245 PMC9739093

[4] National Health Commission of the People’s Republic of China, Disease Prevention and Control Bureau. Report on Nutrition and Chronic Disease status of Chinese Residents (2020). Beijing: People’s Medical Publishing House (2021).

[5] National Health Commission of the People’s Republic of China Notice on the Implementation Plan of the “Year of Weight Management” (2024). Beijing: National Health Commission of the People’s Republic of China official website.

[6] Saintila J Calizaya-Milla YE Carranza-Cubas SP Serpa-Barrientos A Oblitas-Guerrero SM Ramos-Vera C. Body mass index and healthy lifestyle practices among Peruvian university students: a comparative study among academic discipline. Front Nutr. (2024) 11:1361394. doi: 10.3389/fnut.2024.1361394, 38450241 PMC10915028

[7] Bakarman SS Asiri S Bashatha A Syed W Al-Rawi MBA. Evaluation of clinical aspects of obesity among undergraduate healthcare students: a cross-sectional study at King Saud University, Riyadh, Kingdom of Saudi Arabia. J Health Popul Nutr. (2024) 43:159. doi: 10.1186/s41043-024-00651-y, 39402641 PMC11476735

[8] Deng W Yi Z Lee JCK. The demographic variation in nutrition knowledge and relationship with eating attitudes among Chinese university students. Int J Environ Res Public Health. (2024) 21:159. doi: 10.3390/ijerph21020159, 38397650 PMC10888371

[9] Belogianni K Ooms A Lykou A Moir HJ. Nutrition knowledge among university students in the UK: a cross-sectional study. Public Health Nutr. (2022) 25:2834–41. doi: 10.1017/S1368980021004754, 34879889 PMC9991837

[10] Alptekin İM Duman E. Association of dieting self-efficacy, weight management nutrition knowledge, physical activity, and dietary pattern among Turkish adults: a cross-sectional study. BMC Public Health. (2025) 25:3524. doi: 10.1186/s12889-025-24848-w, 41107786 PMC12534975

[11] Sadeghi O Eshaghian N Keshteli AH Askari G Esmaillzadeh A Adibi P. Association of combined healthy lifestyle with general and abdominal obesity. Front Nutr. (2023) 10:1332234. doi: 10.3389/fnut.2023.1332234, 38292697 PMC10824837

[12] Chinese Obesity Task Force. Excerpts from the guidelines for the prevention and control of overweight and obesity in Chinese adults. Chin J Nutr. (2004) 26:1–4. doi: 10.13325/j.cnki.acta.nutr.sin.2004.01.001

[13] Ding C Qiu Y Li W Yuan F Gong W Chen Z . Expert consensus evaluation of the questionnaire on nutrition and health knowledge among Chinese adults. Chin J Health Res. (2022) 51:866–70. doi: 10.19813/j.cnki.weishengyanjiu.2022.06.002 36539859

[14] Yang J Wang S Li H Liu S Chen S Li X . Association between healthy lifestyle score and all-cause mortality among urban and rural older adults in Beijing. Chin J Prev Control Chronic Dis. (2024) 32:412–8. doi: 10.16386/j.cjpccd.issn.1004-6194.2024.06.003

[15] Sun J Guo Y. Association between sedentary behavior and depressive symptoms in adolescents. J Phys Educ. (2024) 31:68–74. doi: 10.16237/j.cnki.cn44-1404/g8.2024.05.014

[16] Chen Y Xu L Mao Y Ye D. Association between lifestyle score and non-alcoholic fatty liver disease. Mod Prev Med. (2024) 51:742–7. doi: 10.20043/j.cnki.MPM.202310283

[17] Ma Y Ma R Li Z Zhang W Ma C. Awareness and determinants of nutrition and health knowledge among adults in four counties of Qinghai Province in 2023. Chin J Health Educ. (2025) 41:144–9. doi: 10.16168/j.cnki.issn.1002-9982.2025.02.009

[18] Spronk I Kullen C Burdon C O’Connor H. Relationship between nutrition knowledge and dietary intake. Br J Nutr. (2014) 111:1713–26. doi: 10.1017/S0007114514000087 24621991

[19] Cooke R Papadaki A. Nutrition label use mediates the positive relationship between nutrition knowledge and attitudes towards healthy eating with dietary quality among university students in the UK. Appetite. (2014) 83:297–303. doi: 10.1016/j.appet.2014.08.039, 25218881

[20] Shook RP Halpin K Carlson JA Davis A Dean K Papa A . Adherence with multiple national healthy lifestyle recommendations in a large pediatric center electronic health record and reduced risk of obesity. Mayo Clin Proc. (2018) 93:1247–55. doi: 10.1016/j.mayocp.2018.04.020, 30060957

[21] Bullo M Garcia-Aloy M Martinez-Gonzalez MA Corella D Fernandez-Ballart JD Fiol M . Association between a healthy lifestyle and general obesity and abdominal obesity in an elderly population at high cardiovascular risk. Prev Med. (2011) 53:155–61. doi: 10.1016/j.ypmed.2011.06.008, 21708186

[22] Oppert JM Bellicha A van Baak MA Battista F Beaulieu K Blundell JE . Exercise training in the management of overweight and obesity in adults: synthesis of the evidence and recommendations from the European Association for the Study of obesity physical activity working group. Obes Rev. (2021) 22:e13273. doi: 10.1111/obr.13273, 34076949 PMC8365734

[23] Morze J Rucker G Danielewicz A Przybylowicz K Neuenschwander M Schlesinger S . Impact of different training modalities on anthropometric outcomes in patients with obesity: a systematic review and network meta-analysis. Obes Rev. (2021) 22:e13218. doi: 10.1111/obr.13218, 33624411 PMC8244024

[24] Hoffmann SW Schierbauer J Zimmermann P Voit T Grothoff A Wachsmuth NB . Effects of interrupting prolonged sitting with light-intensity physical activity on inflammatory and cardiometabolic risk markers in young adults with overweight and obesity: secondary outcome analyses of the SED-ACT randomized controlled crossover trial. Biomolecules. (2024) 14:1029. doi: 10.3390/biom14081029, 39199416 PMC11352707

[25] Audrain-McGovern J Benowitz NL. Cigarette smoking, nicotine, and body weight. Clin Pharmacol Ther. (2011) 90:164–8. doi: 10.1038/clpt.2011.105, 21633341 PMC3195407

[26] Davies A Wellard-Cole L Rangan A Allman-Farinelli M. Validity of self-reported weight and height for BMI classification: a cross-sectional study among young adults. Nutrition. (2020) 71:110622. doi: 10.1016/j.nut.2019.110622, 31837644

