# Supplementary material for: Associations of nutrition knowledge and healthy lifestyle with body mass index status among medical students: a cross-sectional study
Source: Front Nutr. 2026 Sep 10;13:1893421. doi: 10.3389/fnut.2026.1893421 (PMC13600850; doi:10.3389/fnut.2026.1893421)
Supplement: Supplementary file 1 [file Table_1.DOCX]

**Supplementary Table S1. Variable Coding for Influencing Factors**

| Factor | Coding scheme |
| --- | --- |
| Smoking status | Smoker = 0; Non-smoker = 1 |
| Alcohol consumption | Drinker = 0; Non-drinker = 1 |
| Physical activity | < 4 hours/week = 0; ≥ 4 hours/week = 1 |
| Sedentary time | ≥ 8 hours/day = 0;< 8 hours/day = 1 |
| Sleep duration | < 6 hours/day = 0; ≥6 hours/day = 1 |
| Nutrition knowledge | Score < 75 = 0; Score ≥ 75 = 1 |

**Supplementary Table S2. Complete numerical results for the associations of individual lifestyle factors and nutrition knowledge with BMI status**

| Variable | Model 1 | | | | Model 2 | | | |
| --- | --- | --- | --- | --- | --- | --- | --- | --- |
|  | Underweight vs. normal | | Overweight/obesity vs. normal | | Underweight vs. normal | | Overweight/obesity vs. normal | |
|  | 95%*CI* | *P-*value | 95%*CI* | *P-*value | 95%*CI* | *P-*value | 95%*CI* | *P-*value |
| Smoking vs. Non-smoking | 0.658  (0.157-2.750) | 0.566 | 0.178  (0.057-0.554) | 0.003 | 0.441  (0.109-1.777) | 0.249 | 0.107  (0.036-0.320) | <0.001 |
| Drinking vs. Non-drinking | 0.259  (0.182-0.369) | <0.001 | 0.446  (0.325-0.612) | <0.001 | 0.249  (0.176-0.354) | <0.001 | 0.415  (0.304-0.567) | <0.001 |
| Physical activity < 4 hours/week vs. ≥ 4 hours/week | 0.590  (0.422-0.825) | 0.002 | 0.199  (0.133-0.297) | <0.001 | 0.622  (0.451-0.858) | 0.004 | 0.622  (0.451-0.858) | 0.004 |
| Sedentary time ≥8 hours/day vs. < 8 hours/day | 0.457  (0.331-0.630) | <0.001 | 0.742  (0.546-1.008) | 0.056 | 0.510  (0.373-0.697) | <0.001 | 0.855  (0.636-1.149) | 0.299 |
| Sleep duration < 6 hours/day vs. ≥ 6 hours/day | 0.586  (0.427-0.805) | <0.001 | 0.341  (0.243-0.477) | <0.001 | 0.576  (0.420-0.788) | <0.001 | 0.335  (0.240-0.466) | <0.001 |
| Nutrition knowledge <75 points vs. ≥ 75 points | 0.145  (0.103-0.204) | <0.001 | 0.087  (0.060-0.126) | <0.001 | 0.138  (0.099-0.194) | <0.001 | 0.138  (0.099-0.194) | <0.001 |

**Supplementary Table S3. Complete numerical results for the association between HLS and BMI status**

| HLS category | Model 1 | | | | Model 2 | | | |
| --- | --- | --- | --- | --- | --- | --- | --- | --- |
|  | Underweight vs. normal | | Overweight/obesity vs. normal | | Underweight vs. normal | | Overweight/obesity vs. normal | |
|  | 95%*CI* | *P-*value | 95%*CI* | *P-*value | 95%*CI* | *P-*value | 95%*CI* | *P-*value |
| 2 vs 0-1 points | 0.959  (0.577-1.596) | 0.873 | 0.761  (0.465-1.246) | 0.278 | 0.961  (0.580-1.592) | 0.876 | 0.770  (0.476-1.247) | 0.288 |
| 3 vs 0-1 points | 0.222  (0.131-0.376) | <0.001 | 0.222  (0.131-0.376) | <0.001 | 0.230  (0.137-0.388) | <0.001 | 0.209  (0.128-0.341) | <0.001 |
| 4-5 vs 0-1 points | 0.116  (0.061-0.222) | <0.001 | 0.040  (0.019-0.088) | <0.001 | 0.123  (0.065-0.233) | <0.001 | 0.047  (0.022-0.100) | <0.001 |

**Supplementary Table S4. Complete results of exploratory indirect association analysis of nutrition knowledge, HLS, and BMI status**

| BMI comparison | Total association | | Direct association | | Indirect association | | Proportion explained %  (95%*CI)* |
| --- | --- | --- | --- | --- | --- | --- | --- |
|  | 95%*CI* | *P-*value | 95%*CI* | *P-*value | 95%*CI* | *P-*value |  |
| Underweight vs. normal | 0.145  (0.099-0.203) | <0.001 | 0.195  (0.132-0.281) | <0.001 | 0.647  (0.545-0.747) | <0.001 | 22.61  (14.81-32.52) |
| Overweight/obesity vs. normal | 0.086  (0.058-0.121) | <0.001 | 0.115  (0.075-0.165 | <0.001 | 0.554  (0.452-0.656) | <0.001 | 24.12  (16.99-32.22) |

**Supplementary Table S5. Class-specific performance of exploratory random forest models for BMI classification**

| Model | BMI class | Sensitivity | Specificity | Precision | F1 score | Balanced accuracy |
| --- | --- | --- | --- | --- | --- | --- |
| Model A | Normal weight | 0.858 | 0.673 | 0.739 | 0.794 | 0.766 |
| Model A | Underweight | 0.319 | 0.908 | 0.511 | 0.393 | 0.614 |
| Model A | Overweight/obesity | 0.564 | 0.850 | 0.557 | 0.561 | 0.707 |
| Model B | Normal weight | 0.864 | 0.640 | 0.722 | 0.787 | 0.752 |
| Model B | Underweight | 0.000 | 1.000 | - | - | 0.500 |
| Model B | Overweight/obesity | 0.679 | 0.722 | 0.449 | 0.541 | 0.701 |

Note: Model A included sex, age group, individual lifestyle components, and nutrition knowledge. Model B included sex, age group, HLS group, and nutrition knowledge. BMI status was classified as underweight, normal weight, and overweight/obesity. Random forest models were used for exploratory classification and variable importance analysis rather than for clinical prediction. HLS, healthy lifestyle score.

**Supplementary Table S6. Complete variable importance ranking of exploratory random forest models**

| Model | Rank | Variable | MeanDecreaseAccuracy | MeanDecreaseGini |
| --- | --- | --- | --- | --- |
| Model A | 1 | Nutrition knowledge ≥75 vs. <75 points | 80.675 | 67.539 |
|  | 2 | Sleep ≥6 h/day vs. <6 h/day | 22.282 | 13.160 |
|  | 3 | Non-drinking vs. drinking | 22.189 | 17.566 |
|  | 4 | Sedentary time <8 h/day vs. ≥8 h/day | 20.839 | 16.098 |
|  | 5 | Physical activity ≥4 h/week vs. <4 h/week | 18.233 | 13.431 |
|  | 6 | Non-smoking vs. smoking | 11.594 | 6.561 |
|  | 7 | Sex (female vs. male) | 7.791 | 11.273 |
|  | 8 | Age >22 vs. 18–22 years | 4.054 | 5.930 |
|  | 9 | Age <18 vs. 18–22 years | -8.922 | 1.501 |
| Model B | 1 | Nutrition knowledge ≥75 vs. <75 points | 37.326 | 50.519 |
|  | 2 | HLS = 4-5 vs. 0-1 | 18.495 | 13.813 |
|  | 3 | HLS = 3 vs. 0-1 | 14.044 | 7.610 |
|  | 4 | Sex (female vs. male) | 10.986 | 4.960 |
|  | 5 | Age >22 vs. 18-22 years | 10.251 | 3.929 |
|  | 6 | HLS = 2 vs. 0-1 | 9.340 | 9.240 |
|  | 7 | Age <18 vs. 18-22 years | -3.897 | 1.094 |

Note: Variables were ranked according to MeanDecreaseAccuracy within each model. MeanDecreaseAccuracy reflects the decrease in classification accuracy after permutation; MeanDecreaseGini reflects the decrease in node impurity. Results indicate exploratory variable importance rather than causal importance.
