# Supplementary material for: Associations of nutrition knowledge and healthy lifestyle with body mass index status among medical students: a cross-sectional study
Source: Front Nutr. 2026 Sep 10;13:1893421. doi: 10.3389/fnut.2026.1893421 (PMC13600850; doi:10.3389/fnut.2026.1893421)
Supplement: Supplementary file 2 [file Table_2.DOCX]

**Appendix: University Student Lifestyle and Nutrition Knowledge Questionnaire**

This questionnaire aims to assess lifestyle behaviors, nutrition knowledge, and related health information among university students. Please answer the following questions truthfully.

Your participation is entirely voluntary, and you may withdraw at any time without penalty.

The information you provide will be kept strictly confidential and used solely for research purposes; your identity will not be disclosed.

By completing this questionnaire, you indicate that you have read and understood the study information and agree to participate.

**□ I have read and agree to participate in this study.**

**Part 1: Self-designed survey on personal basic information and lifestyle**

| Question | Options / Description |
| --- | --- |
| A1. Name |  |
| A2. Gender | □ Male □ Female |
| A3. Ethnicity | □ Han □ Other |
| A4. Date of Birth | Year ____ Month ____ Day ____ |
| A5. Height and Weight | Height (cm): ____ Weight (kg): ____ |
| B1. Did you smoke in the past 30 days? | □ No □ Every day □ Occasionally |
| B2. Have you consumed the following alcoholic beverages in the past 12 months? | □ Chinese Baijiu  □ Yellow rice wine  □ Rice wine  □ Beer  □ Wine  □ Other |
| B3. Over the past year, how much time did you spend on moderate or high-intensity activities on average each week? | Hours ___ Minutes ___ |
| B4. How long do you typically spend in a static state in a day? (This includes the cumulative time spent sitting, lying down, or reclining, covering all static activities such as sitting for work, studying, reading, watching TV, using the computer, and resting, but excluding sleep time.) | Hours ___ Minutes ___ |
| B5. How long is the total duration of your sleep (including naps) in a single day? | Hours ___ Minutes ___ |

**Part 2: The Nutrition and Health Knowledge Questionnaire for Chinese Adults Aged 18-64 (Developed by the Institute of Nutrition and Health, Chinese Center for Disease Control and Prevention)**

| Q1. According to the Dietary Guidelines for Chinese Residents, how should the following foods be consumed? | |
| --- | --- |
| \| More \| Moderate \| Less \| Indifferent \| Don’t know; Vegetables \| \| \| \| \|; Fruits \| \| \| \| \|; Dairy \| \| \| \| \|; Soy products \| \| \| \| \|; Meat \| \| \| \| \|; Processed meat \| \| \| \| \|; Sugary foods \| \| \| \| \| | |
| Q2. What is the recommended maximum daily salt intake for healthy adults? | □ 5g □ 10g □ 15g □ 20g □ Don’t know |
| Q3. What is the recommended maximum daily intake of added sugar? | □ 10g □ 20g □ 30g □ 50g □ Don’t know |
| Q4. What is the recommended daily amount of cooking oil? | □ 10-25g □ 25-30g □ 30-50g □ 50-75g |
| Q5. Which of the following statements about vegetables and fruits is correct? (Multiple choice) | □Include vegetables with every meal  □Eat more dark-colored vegetables; they should make up at least half of your total vegetable intake  □Eat fruit every day  □Vegetables and fruits can substitute for one another  □Don’t know |
| Q6. Which of the following statements about balanced diet is correct? (Multiple choice) | □For adults, no single food can provide all the necessary nutrients for the human body  □Daily meals should include: cereals and tubers; vegetables and fruits; meat, poultry, fish, eggs, and dairy; and soy products and nuts  □By combining coarse and refined grains, balancing plant‑based and animal‑based foods, and varying food colors, it becomes easier to achieve dietary diversity  □Aim to eat more than 12 different foods on average each day, and over 25 different foods each week  □Don’t know |
| Q7. Which of the following foods is best for calcium supplementation? | □ Milk □ Egg □ Soybean □ Meat |
| Q8. Compared with refined grains, coarse grains have which characteristics? (Multiple choice) | □Provides higher amounts of B vitamins  □More likely to raise blood glucose levels  □Provides greater amounts of minerals  □Provides higher amounts of dietary fiber  □Don’t know |
| Q9. Which of the following foods are rich in iron and easily absorbed by the human body? (Multiple choice) | □Duck blood  □Pork liver  □Spinach  □Beef  □Don’t know |
| Q10. To supplement vitamin A, which foods should be consumed? (Multiple choice) | □Napa cabbage  □Chicken liver  □Carrot  □Orange  □Don’t know |
| Q11. Which of the following foods contains more oil and salt? | □Walnut  □Potato chips  □Roasted sunflower seeds  □Milk  □Don’t know |
| Q12. Which type of meat should be prioritized to prevent cardiovascular diseases? | □Fish  □Pork  □Lamb/mutton  □Beef  □Don’t know |
| Q13. Which of the following statements is correct? (Multiple choice) | □Excessive salt intake can increase the risk of hypertension and stroke  □Excessive salt intake can increase the risk of stomach cancer.  □Drinking too many sugar‑sweetened beverages can increase the risk of dental caries  □Drinking too many sugar‑sweetened beverages can increase the risk of obesity and diabetes  □Don’t know |
| Q14. Which of the following statements is correct? (Multiple choice) | □Eating fewer refined staple foods can help prevent diabetes  □Eating more animal fats helps prevent stroke  □Eating fewer processed meat products can help prevent cancer  □Eating more leafy green vegetables can help prevent cardiovascular disease  □Don’t know |
| Read the food labels of the two products below and answer questions 15 and 16. Product 1 Ingredients: Fresh milk Nutrition Facts (\| Per100ml \| NRV%; Energy \| 309kJ \| 4%; Protein \| 3.6g \| 6%; Fat \| 4.4g \| 7%; Carbohydrates \| 5.0g \| 2%; Sodium \| 58mg \| 3%; Calcium \| 120mg \| 15%) \| Product 2 Ingredients: Water, fresh milk, sugar, whole milk powder, high-fructose corn syrup, food additives (citric acid, lactic acid, L-malic acid, sodium cyclamate, acesulfame potassium, aspartame) Nutrition Facts (\| Per100ml \| NRV%; Energy \| 128kJ \| 2%; Protein \| 1.0g \| 2%; Fat \| 1.4g \| 2%; Carbohydrates \| 3.5g \| 1%; Sodium \| 80mg \| 4%) | |
| Q15. Please select which product has higher protein content per 100 ml? | □Product 1  □Product 2  □Both have the same content  □ Don’t know |
| Q16. Which of the two products belongs to the category of dairy products? | □Product 1  □Product 2  □Both of product 1 and 2  □Neither of product 1 and 2 |
| Q17. Please estimate the weight of the following foods. | |
| \| 10g \| 20g \| 50g \| 100g \| 200g \| Don’t know;  \| \| \| \| \| \|;  \| \| \| \| \| \|;  \| \| \| \| \| \|;  \| \| \| \| \| \| | |
| Q18. Which of the following lunch combinations is more nutritionally balanced? | □‌Mixed Bean Rice + Braised Beef with Tomatoes + Stir-Fried Spinach  □Steamed Mantou + Sautéed Shredded Potatoes in Vinegar Sauce + Stir-Fried Zucchini  □Chicken Burger + French Fries + Drink  □Braised Spare Ribs + Braised Fish in Soy Sauce + Stir-Fried Chinese Greens (Youcai)  □Don’t know |
| Q19. If an adult’s BMI is 26.1 kg/m², his/her body weight is classified as: | □ Normal  □ Underweight  □ Overweight  □ Obese  □ Don’t know |
| Q20. Which of the following practices help maintain a healthy weight? (Multiple choice) | □Manage your diet and avoid overeating  □Engage in moderate physical activity daily  □Learn to read food labels and reduce intake of high-calorie foods  □Pay attention to food energy content and balance meals wisely  □Don’t know |
| Q21. To avoid food waste, which of the following practices are correct? (Multiple choice) | □Buy ingredients in appropriate quantities to avoid excess  □Avoid over-processing food to minimize kitchen waste  □Eat the rest food even if you are full  □Order meals based on the number of people when dining out, avoiding extravagance  □Don’t know |
| Q22. Which of the following statements about food safety and hygiene are correct? (Multiple choice) | □Use separate cutting boards for raw meat and cooked meat  □After washing the knife that has cut raw meat with water, it can be used to cut cooked meat  □ The use of serving spoons and chopsticks is not necessary when dining at home  □Always use serving spoons/chopsticks when dining with friends  □Don’t know |

Note: This English version is a translation of the original Chinese questionnaire used in the study.
